# Association between metabolic syndrome and pelvic organ prolapse severity

**DOI:** 10.1007/s00192-014-2468-9

**Published:** 2014-07-22

**Authors:** A. Rogowski, P. Bienkowski, D. Tarwacki, E. Dziech, J. Samochowiec, M. Jerzak, W. Baranowski

**Affiliations:** 1 Department of Gynecology and Oncological Gynecology, Military Institute of Medicine, 28 Szaserow St., 04-141, Warsaw, Poland; 2 Department of Pharmacology, Institute of Psychiatry and Neurology, Warsaw, Poland; 3 Department of Psychiatry, Pomeranian Medical University, Szczecin, Poland

**Keywords:** Pelvic organ prolapse, Metabolic syndrome, Triglycerides

## Abstract

**Introduction and hypothesis:**

As in the case of cardiovascular and metabolic diseases, the prevalence of pelvic organ prolapse (POP) has been rising with the increasing proportion of elderly women in the population. The purpose of the present cross-sectional study was to evaluate the components of metabolic syndrome (MS) in urogynecological patients with a variable POP severity.

**Methods:**

The MS risk factors (elevated waist circumference, elevated triglycerides, decreased high-density lipoprotein cholesterol, elevated blood pressure, hyperglycemia) were assessed in 100 women who were referred to our urogynecological center with pelvic floor disorders (PFD). POP was evaluated with the Pelvic Organ Prolapse Quantification system (POP-Q).

**Results:**

The *χ* ^2^ test revealed that the diagnosis of MS and the presence of elevated triglycerides increased with the overall POP-Q stage. The other MS risk factors were not significantly associated with the overall POP-Q stage. MS and elevated triglycerides were predictors of the POP-Q stage ≥III [odds ratio (OR) 3.5, 95 % confidence interval (CI) 1.5–8.2 for MS and OR 3.4, 95 % CI 1.4–8.2 for elevated triglycerides, *p* < 0.01].

**Conclusions:**

The diagnosis of MS and the presence of elevated triglycerides may be associated with the severity of POP in urogynecological patients. Longitudinal studies are required to assess whether the MS risk factors might predict the progression of POP and whether elimination of the risk factors might improve the prognosis in POP patients.

## Introduction

Pelvic organ prolapse (POP) imposes a significant medical, psychological, and economic burden on individual patients and societies [1–3]. The prevalence of POP has been rising with the increasing proportion of elderly women in the population. It has been reported that about 50 % of parous women develop POP symptoms [4] and the lifetime risk of POP surgery is 11 % [5]. The cause of POP is considered to be multifactorial [3, 6]. Vaginal childbirth, ageing, increased body mass index (BMI), levator ani avulsions, and variations in shape of the bony pelvis may all constitute risk factors for POP [1, 3, 7].

Some of the above risk factors increase the risk of developing POP but not its severity once patients become symptomatic. For example, although obesity increases the risk of developing vaginal prolapse [1], BMI is not correlated with POP severity assessed with the Pelvic Organ Prolapse Quantification system (POP-Q) [8, 9]. Washington et al. [10] reported no significant difference in stage ≥II prolapse between obese and nonobese patients seeking care for pelvic floor disorders (PFD). In line with the above, no correlation was observed between BMI and the overall POP-Q stage in a retrospective study on women with symptomatic prolapse presenting to an outpatient urogynecological center [9].

Most studies showing a positive correlation between obesity and POP focused on the theory of increased abdominal pressure. According to the theory, chronic obesity-related increase in abdominal pressure stresses the pelvic floor and leads to structural damage and/or neurological dysfunction predisposing to POP [1, 6]. Obesity-related metabolic and vascular complications [11, 12] have received relatively little attention in the discussion on the pathophysiology of POP. Decades of research have revealed abdominal obesity to be a precursor and crucial aspect of metabolic syndrome (MS), a cluster of interrelated risk factors for cardiovascular disorders, which occur together more often than by chance alone [13–15]. The recently updated criteria for clinical diagnosis of MS include elevated waist circumference, elevated triglycerides, reduced high-density lipoprotein cholesterol, elevated blood pressure, and hyperglycemia [13].

A possible relationship between MS and POP has not been addressed in much detail. In their single cross-sectional study, Kim et al. [16] analyzed correlations between MS and the severity of POP symptoms assessed with the Pelvic Floor Distress Inventory 20 (PFDI-20) in Korean women recruited during a routine medical screening. The PFDI-20 is a condition-specific questionnaire consisting of 20 questions divided into 3 subscales: the Pelvic Organ Prolapse Distress Inventory 6 (POPDI-6), Colorectal-Anal Distress Inventory 8 (CRADI-8), and Urinary Distress Inventory 6 (UDI-6) [17]. In the study by Kim et al. [16], the presence of MS was associated with the total PFDI-20 score and the CRADI-8, UDI-6, and POPDI-6 subscores. Among the MS criteria, only elevated waist circumference and elevated triglycerides were significant risk factors for POP symptoms. It should be remembered that the assessment of POP symptoms in the latter study was based on the patient’s self-report, performed in a primary care setting, and limited to Asian women [16]. It remains to be established whether a similar relationship exists between MS and the severity of POP diagnosed on the basis of physical examination and the POP-Q staging in urogynecological patients of Caucasian origin.

The purpose of the present pilot, cross-sectional study was to evaluate the MS risk factors in urogynecological patients with different POP severity. Given the data reported by Kim et al. [16], we hypothesized that patients with a greater overall POP-Q stage are more likely to be diagnosed with MS and that some components of MS may be significant risk factors for more severe POP.

## Materials and methods

### Study protocol

The study was carried out in accordance with the Declaration of Helsinki of the World Medical Association. The study protocol was approved by the Ethics Committee for Human Studies of the Military Institute of Medicine, Warsaw, Poland. All participants signed an informed consent form after study procedures had been fully explained.

**Fig. 1:**
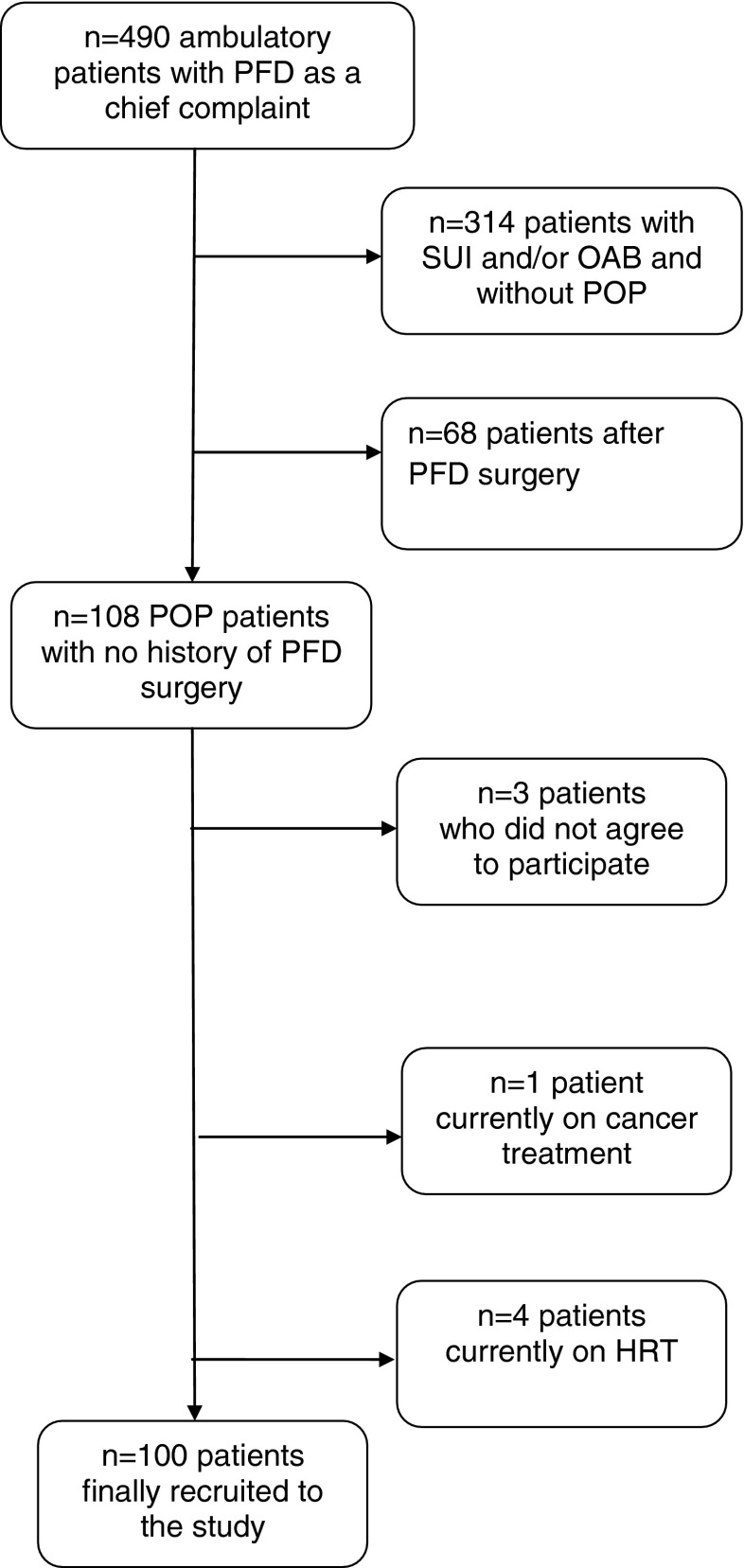
Flow chart of patients’ recruitment

Women (*n* = 490) who were referred to the Outpatient Clinic of the Department of Gynecology in 2012 with PFD as a chief complaint were screened for eligibility [9, 10; for details, see Fig. 1]. A total of 108 patients diagnosed with POP with the POP-Q staging system were considered potential participants. Exclusion criteria comprised a prior bariatric or PFD surgery and having a serious medical condition which might render the interpretation of results difficult (e.g., hepatic or renal insufficiency, cancer treatment, a genetic connective tissue disease, alcohol dependence). We also excluded women who had received current or recent hormone replacement therapy (HRT) to avoid the likely association between HRT and lipid profiles [18].

Study methods and definitions conformed to the standards jointly recommended by the International Urogynecological Association and the International Continence Society (IUGA/ICS) [19]. The pelvic examination was performed with the patient in the dorsal lithotomy position. The POP-Q system was used to quantify the severity of POP at a maximum Valsalva strain [8]. Stage 0 implies normal anatomy, without prolapse in the vaginal compartment. In patients with stage I prolapse, the leading edge of the prolapse was >1 cm above the level of the hymen. For stage II, the most distal portion of the prolapse was within 1 cm of the hymen. In patients with stages III and IV, the leading edge of the prolapse protruded the hymen. In those at stage IV, the prolapse protruded the greatest distance [total vaginal length (tvl) − 2] cm beyond the plane of the hymen [8, 9, 20]. The patients were screened for stress urinary incontinence (SUI) symptoms using the Stamey Incontinence Score [21]. Those scoring ≥1 were considered to be SUI positive [20]. Overactive bladder (OAB) symptoms were assessed by using questions selected from the PFDI [22] as described by Rogowski et al. [20].

The final study group included 100 Caucasian women with overall POP-Q stage I–IV (stage I, *n* = 22, stage II, *n* = 21, stage III, *n* = 46, stage IV, *n* = 11). These patients were screened for the presence of MS risk factors as reported by other authors [11, 14, 16]. For each patient, the MS criteria were assessed in the Outpatient Clinic of the Department of Gynecology. Anthropometric measurements (weight, height) were made with the patients wearing light clothes with no shoes. BMI was calculated as weight (in kg) divided by height (in square meters). Waist circumference was measured on minimal inspiration with a tape with 0.5-cm accuracy from the narrowest point between the lower rib border and the iliac crest. Blood pressure was taken at least twice, with the patient in a sitting position, after a 10-min period of relaxation. Blood samples were collected in the morning after a 12-h fast and sent to the laboratory of the Military Institute of Medicine. Commercial enzymatic tests were used to determine fasting glucose, triglycerides, and high-density lipoprotein cholesterol [11, 14, 16].

The patients were diagnosed with MS if any three (or more) of the following five risk factors were present: (1) abdominal obesity, i.e., waist circumference ≥80 cm, (2) hyperglycemia, i.e., fasting glucose ≥100 mg/dl (or treatment for elevated glucose), (3) hypertriglyceridemia, i.e., fasting triglycerides ≥150 mg/dl (or treatment for elevated triglycerides),(4) high-density lipoprotein cholesterol <50 mg/dl (or treatment for reduced high-density lipoprotein cholesterol), and (5) elevated blood pressure, i.e., systolic pressure ≥130 mmHg and/or diastolic pressure ≥85 mmHg (or antihypertensive drug treatment in patients with a history of hypertension) [13, 14].

### Statistics

Sociodemographic and clinical parameters were expressed as means (±SD) or proportions (*n*/*N*). The subgroups of patients with a different POP-Q stage were compared with the one-way analysis of variance (ANOVA) or the *χ* ^2^ test. Odds ratios (ORs) with 95 % confidence intervals (CI) were used to estimate the strength of associations between the MS risk factors and the POP-Q stage. The number of subjects with POP-Q stage IV was relatively small, and thus for some statistical analyses the patients were pooled into two groups (POP-Q stage I–II and POP-Q stage III–IV). ORs with 95 % CI were calculated for the association between the MS risk factors and the POP-Q stage ≥III.

A probability level (*p*) less than 0.05 was considered significant. All statistical analyses were performed using the Statistica 5.0 software package (StatSoft, Tulsa, OK, USA).

## Results

Figure 1 shows the detailed flow chart of patients’ recruitment. Table 1 shows basic sociodemographic and clinical characteristics of the study participants with different POP-Q stages (I–IV). The four groups did not differ in age, BMI, smoking status, gravidity, parity, and OAB symptoms. Similarly, there were no differences in the above parameters when the patients with POP-Q stage ≤II (stage I–II) were compared with those with stage ≥III (stage III–IV; all *p* > 0.05).

**Table 1:** Sociodemographic and clinical characteristics of patients (*n* = 100) with different POP-Q stages

Parameter	Stage I	Stage II	Stage III	Stage IV	Statistics
Age (years)^a^	60.1 ± 14.2	60.3 ± 9.0	64.7 ± 7.9	63.1 ± 8.9	F_(3,96)_ = 1.5, *p* = 0.2
BMI (kg/m^2^)^a^	27.1 ± 3.9	28.9 ± 5.3	28.5 ± 5.1	26.4 ± 4.8	F_(3,96)_ = 1.0, *p* = 0.4
Current smokers^b^	1/22	3/21	2/46	1/11	*χ* ^2^ (3) = 2.5, *p* = 0.4
Gravidity^a^	2.2 ± 1.2	2.5 ± 1.0	2.5 ± 1.5	2.9 ± 1.8	F_(3,96)_ = 0.6, *p* = 0.6
Parity^a^	1.9 ± 0.9	2.2 ± 1.0	1.9 ± 1.2	2.3 ± 1.3	F_(3,96)_ = 0.6, *p* = 0.6
Postmenopausal status^b^	13/22	19/21	44/46	11/11	*χ* ^2^ (3) = 20.0, *p* = 0.0001
SUI symptoms^b^	19/22	14/21	17/46	5/11	*χ* ^2^ (3) = 16.4, *p* = 0.0009
OAB symptoms^b^	8/22	9/21	16/46	3/11	*χ* ^2^ (3) = 0.8, *p* = 0.9

*POP-Q* Pelvic Organ Prolapse Quantification system [8]

^a^Mean ± SD

^b^ *n*/*N*

The *χ* ^2^ test showed a significant difference between the four groups in the proportion of postmenopausal women. The above difference was mainly due to the fact that more premenopausal patients were classified as stage I. The *χ* ^2^ test also revealed significant differences between the four groups in the proportion of women reporting SUI symptoms. The proportion of SUI-positive patients tended to decrease with increasing POP-Q stage (Table 1).

The prevalence of MS and its components in the groups with different POP-Q stages is shown in Table 2. The *χ* ^2^ test indicated that the prevalence of elevated waist circumference, reduced high-density lipoprotein cholesterol, elevated blood pressure, and elevated fasting glucose did not differ between the four groups. The proportion of patients with elevated triglycerides increased with increasing POP-Q stage. Similarly, the proportion of women meeting the criteria for MS increased with increasing POP-Q stage (Table 2). The calculation of ORs with 95 % CI confirmed that elevated triglycerides and the diagnosis of MS were significant predictors of POP-Q stage ≥III (Table 3).

**Table 2:** MS and its components in patients with different POP-Q stages

Risk factor	Stage I	Stage II	Stage III	Stage IV	Statistics
Elevated waist circumference	15/22	19/21	39/46	8/11	*χ* ^2^ (3) = 4.5, *p* = 0.2
Elevated triglycerides	6/22	4/21	24/46	5/11	*χ* ^2^ (3) = 8.3, *p* = 0.04
Reduced high-density lipoprotein cholesterol	1/22	4/21	10/46	1/11	*χ* ^2^ (3) = 3.8, *p* = 0.3
Elevated blood pressure	14/22	17/21	28/46	5/11	*χ* ^2^ (3) = 4.4, *p* = 0.2
Elevated fasting glucose	4/22	6/21	19/46	4/11	*χ* ^2^ (3) = 3.9, *p* = 0.3
MS^a^	3/22	8/21	26/46	5/11	*χ* ^2^ (3) = 11.4, *p* = 0.009

Data presented as *n*/*N*

*POP-Q* Pelvic Organ Prolapse Quantification system [8]

^a^The presence of any 3 (or more) of 5 risk factors constituted a diagnosis of MS [13]

**Table 3:** MS and its components as predictors of increased POP-Q stage

Risk factor	OR	95 % CI	*p* value
Elevated waist circumference	1.2	0.4–3.5	*p* = 0.7
Elevated triglycerides	3.4	1.4–8.2	*p* = 0.006
Reduced high-density lipoprotein cholesterol	1.8	0.6–5.7	*p* = 0.3
Elevated blood pressure	0.5	0.2–1.2	*p* = 0.1
Elevated fasting glucose	2.2	0.9–5.4	*p* = 0.1
MS	3.5	1.5–8.2	*p* = 0.005

43 patients were classified as POP-Q stage ≤II and 57 patients were classified as POP-Q stage ≥III; univariate regression analysis was used to predict stage ≥III

*POP-Q* Pelvic Organ Prolapse Quantification system [8]

Since the four groups (POP-Q stage I–IV) differed in the number of postmenopausal women, a separate analysis was performed to study the association between MS and POP-Q stage after the exclusion of 13 premenopausal patients. The numbers of stage I and stage IV patients were relatively low, and thus the remaining postmenopausal subjects were pooled into two groups (postmenopausal/POP-Q stage I–II vs postmenopausal/POP-Q stage III–IV). The proportion of women with MS was significantly higher in the postmenopausal/stage III–IV group (31/55) as compared to the postmenopausal/stage I–II group [11/32; *χ* ^2^ (1) = 3.9, *p* < 0.05]. The proportion of women with elevated triglycerides was also significantly higher in the postmenopausal/stage III–IV group (29/55) in comparison with the postmenopausal/stage I–II group [7/32; *χ* ^2^ (1) = 7.9, *p* < 0.01]. The postmenopausal/stage III–IV and postmenopausal/stage I–II subjects did not differ in the prevalence of the other MS components (*p* > 0.05).

## Discussion

To the best of our knowledge, this is the first study showing that MS and some of its components may be associated with the severity of POP assessed with the POP-Q staging system in urogynecological patients seeking help for PFD. The prevalence of MS in the POP-Q stage III–IV group was twice as high as the prevalence found in the POP-Q stage I–II group. Notably, the prevalence of MS observed in the whole study group (42 %) was similar to that reported recently for a representative sample of Polish women recruited in the community (from 39 to 44 % depending on the MS definition) [23].

The associations between MS, triglycerides, and POP-Q stage remained significant regardless of whether the whole group or the postmenopausal women were included in the statistical analyses. Interestingly, only the diagnosis of MS and the presence of elevated triglycerides, but not the other MS components, were significantly associated with POP-Q stage. In line with previous reports [9, 10], neither BMI nor waist circumference was correlated with POP-Q stage in the present study. Thus, increased BMI and obesity may be risk factors for POP but not necessarily for POP severity once patients become symptomatic [9, 10].

A relationship between MS risk factors and POP has not been addressed in much detail in the gynecological literature. Our findings closely correspond with the results of the report by Kim et al. [16] showing that the presence of MS was associated with the severity of POP symptoms assessed with the POPDI-6. Among the MS criteria, only elevated waist circumference and elevated triglycerides were related to POP symptoms. The assessment of POP symptoms in the latter study was based on the patient’s self-report, performed in a primary care setting, and limited to Asian women [16]. Hence, it seems that the association between MS, elevated triglycerides, and the severity of POP is not specific to a particular technique of assessment (the questionnaire in primary care vs physical examination in urogynecological patients) or the patient’s ethnicity (Asian vs Caucasian).

MS may increase the severity of POP through different, but not mutually exclusive, mechanisms. It has been repeatedly reported that patients with MS show microvascular pathological conditions secondary to endothelial dysfunction and atherosclerosis. MS also increases the risk of prothrombotic and proinflammatory states [12, 14, 15, 24]. The above pathological conditions may be associated with remodeling of pelvic floor connective tissues leading to aberrations in synthesis and/or degradation of collagen and elastin fibers [6, 25].

The present results suggest that triglycerides are specifically involved in POP pathology. The prevalence of elevated triglycerides in our patients (stage ≤II 23.2 %, stage ≥III 50.9 %) was clearly higher than the prevalence found (12.5 %) in a representative sample of Polish women (age 56.4 ± 11.8 years, BMI 27.9 ± 5.1 kg/m^2^) recruited in the community [23]. The role of triglycerides in POP has not been addressed in systematic preclinical or clinical studies. Hypertriglyceridemia is a common lipid metabolic disorder, often of genetic origin, which may be modeled in laboratory animals [26, 27]. If triglycerides play a specific role in POP, more POP symptomatology may be expected in women with primary hypertriglyceridemia [27] and in animal models of hypertriglyceridemia [26, 28]. The above hypotheses can be addressed in further experimental and clinical studies.

In the present study, the self-reported SUI symptoms were negatively correlated with the presence of MS. Interestingly, Kim et al. [16] showed a positive correlation between MS and various micturition disorders evaluated with the UDI-6 in Asian women. One should be aware that the real prevalence of SUI in patients with POP-Q stage ≥III is probably higher than might be inferred from the self-report. It has been shown that POP may obstruct the urethra and advanced POP is often associated with occult urinary incontinence [29]. Further studies should be conducted to evaluate possible associations between MS and urinary incontinence in patients with and without POP.

The present study involves some limitations. We included a relatively small sample of patients recruited in one urogynecological center localized in an urban area. The study was cross-sectional in nature and does not enable a judgment about casual relationships or validity of the MS risk factors for predicting the progression of POP. One should also remember that MS and elevated triglycerides may not be independent predictors of POP. Further studies on larger samples are needed to replicate the present findings and to evaluate whether MS and elevated triglycerides are independent risk factors for POP severity.

Given the significance of POP for individual patients and societies [1, 2], longitudinal studies are needed to evaluate whether the presence of MS alters the prognosis in POP patients. If yes, it would be of critical importance to evaluate whether patients’ counseling and/or pharmacotherapy aimed at eliminating the MS risk factors could inhibit the progression of POP.

## Abbreviations

BMI: Body mass index
CI: Confidence interval
CRADI-8: Colorectal-Anal Distress Inventory 8
HRT: Hormone replacement therapy
MS: Metabolic syndrome
OAB: Overactive bladder
OR: Odds ratio
PFD: Pelvic floor disorders
PFDI-20: Pelvic Floor Distress Inventory 20
POP: Pelvic organ prolapse
POPDI-6: Pelvic Organ Prolapse Distress Inventory 6
POP-Q: Pelvic Organ Prolapse Quantification
SUI: Stress urinary incontinence
tvl: Total vaginal length
UDI-6: Urinary Distress Inventory 6
